# The Combined Use of the Temporoparietal Fascia Graft and Polycaprolactone Nasal Sheets in Iatrogenic Nasal Septal Perforation Repair

**DOI:** 10.1007/s00266-025-05088-0

**Published:** 2025-08-07

**Authors:** Murat Yaşar

**Affiliations:** Department of Otorhinolaryngology, Head and Neck Surgery, Faculty of Medicine, Kastamonu University, 37200 Kastamonu, Turkey

**Keywords:** Iatrogenic, Endoscopic, Nasal septum, Perforation

## Abstract

**Objective:**

Medium- and large-sized iatrogenic nasal septal perforations are difficult to repair. Many techniques using local flaps with or without interposition grafts are used to repair septum perforation. The aim of this study was to describe our surgical technique for repairing iatrogenic nasal septal perforations with medium sized (1–2 cm) and large sized (> 2 cm).

**Methods:**

We retrospectively reviewed 15 patients with medium- and large-sized iatrogenic nasal septal perforations treated with a sandwich technique (temporoparietal fascia and polycaprolactone nasal sheet as an interposition graft) by an endoscope-assisted intranasal approach at the Kastamonu Training and Research Hospital from September 2021 to September 2024. Clinical follow-up periods were between 3 and 28 months in duration (mean 12 months).

**Results:**

Fifteen patients, nine men and six women, were operated on using this technique. A mean follow-up time is 12 months. Complete closure was achieved in 13 of 15 patients (86.6%). No complications were also occurred in either the early or late postoperative periods.

**Conclusion:**

An endoscopic sandwich technique that uses a temporalis fascia polycaprolactone nasal sheet combined as an interpositioning graft to repair iatrogenic nasal septal perforations is described. The transnasal endoscopic sandwich technique for repairing of medium-sized and large-sized iatrogenic nasal septal perforations has a high success rate and is relatively easy to perform.

**Level of Evidence IV:**

This journal requires that authors assign a level of evidence to each article. For a full description of these Evidence-Based Medicine ratings, please refer to the Table of Contents or the online Instructions to Authors www.springer.com/00266.

## Introduction

Nasal septal perforations (NSP) represent a loss of the three-layered wall of the nasal septum that emerges due to iatrogenic causes and for reasons such as nasal trauma, neoplasias, granulomatous diseases, and the use of inhaled irritants [1, 2]. NSP has a significant impact on quality of life and is an important source of morbidity. It can give rise to symptoms such as nasal obstruction, in particular, nasal crusting, a whistling sound when breathing, nasal discharge, epistaxis, olfactory disorder, and headache [2].

Numerous surgical techniques have been reported for the repair of NSP, with extra- and intra-nasal approaches such as the closure of the mucoperichondrium by direct suturing and local intranasal flap use, interposition grafting with both synthetic and autologous graft material, alloplasty, fascia grafts, acellular human dermal allograft, and bone and cartilage grafts. However, success rates still range between 75 and 96%. The reperforation rate is also between 12 and 48%. Although small perforations can be closed with the majority of these techniques, the closure of medium-sized and large-diameter perforations is more difficult due to the absence of a standard approach and technique selection [2–11]. The search for novel surgical NSP repair techniques is, therefore, still ongoing.

Polycaprolactone (PCL) is one of the U.S. Federal Drug Administration-approved biodegradable, slowly absorbed, linear polyester biomaterials used in tissue engineering. Three-dimensional printed PCL nasal mesh, introduced as an alternative to autologous implants, has recently been demonstrated to possess adequate mechanical support, thinness, and surgical manipulability [12–14]. We encountered no previous studies of the use of the PCL nasal sheet in nasal septal perforation. The purpose of this study was to retrospectively examine cases of medium-sized (1–2 cm) and large-sized (> 2 cm) iatrogenic NSP repair using a three-layer interposition graft consisting of a combined temporoparietal fascia (TPF) and PCL nasal sheet (Bloocell® Bioscaffold; Bloocell nasal sheet) (for subsequent use as a sandwich graft) and evaluating the type of application and outcomes of the endoscopic sandwich graft technique performed.

## Materials and Methods

**Fig. 1:**
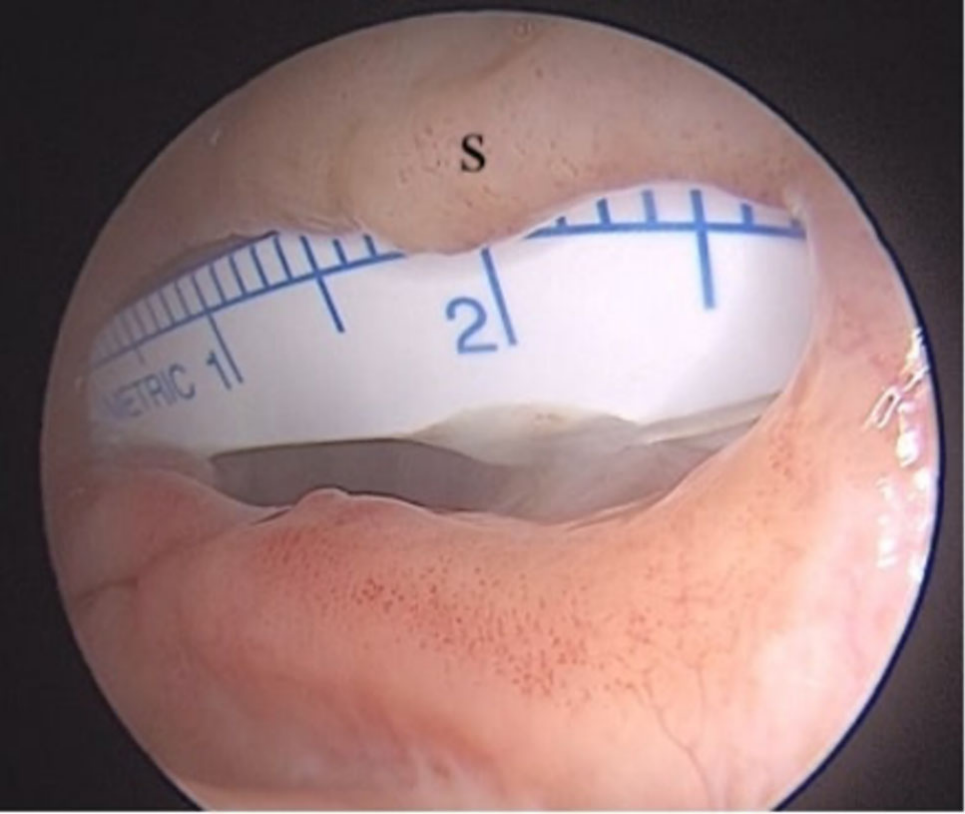
Perforation located on the inferior–middle of the septum with anterior–posterior a diameter of 2.7 cm. *S* Septum

This retrospective study was performed in accordance with the principles of the Declaration of Helsinki. Approval of the study was received from Kastamonu University Clinical Research Ethics Committee (Date: 26.09.2024 Decision No: 2024-KAEK-62). Data were obtained from retrospective examinations of 15 patients operated due to medium- and large-sized NSP at the Kastamonu Training and Research Hospital Ear, Nose, and Throat Clinic, Kastamonu, between September 2021 and September 2024 (Fig. 1). All patients were operated under general anesthesia by the same surgeon. The etiological cause in all cases was previous nasal septum surgery. No saddle nose deformity was present in any patient. The study data included demographic characteristics, current symptoms and findings, the perforation dimension, recurrence, and clinical follow-ups between three and 28 months in duration (mean 12 months).

### Surgical Technique

**Fig. 2:**
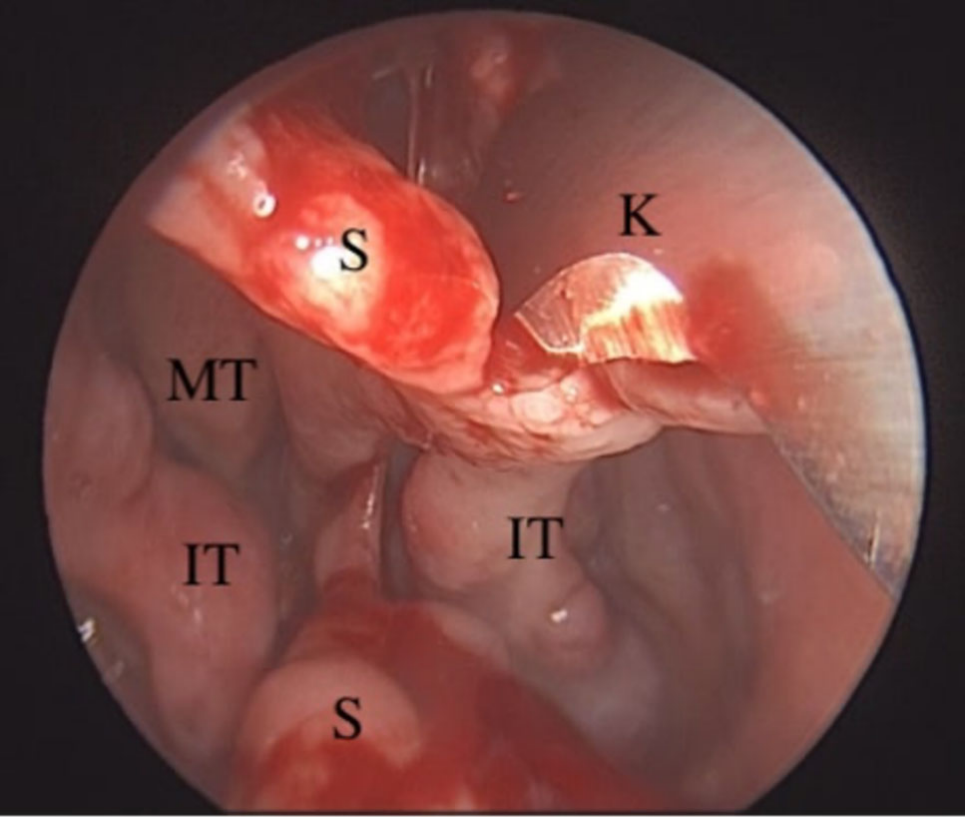
The flap at the perforation edge was elevated with a # 15 scarpel for preventing laceration. *S* Septum, *K* Knife, *MT* Middle turbinate, *IT* İnferior turbinate

The nasal mucosa was first decongested with merocel tampon soaked in 1:100,000 epinephrine. Prior to the incision, 1:100,000 epinephrine was infiltrated around the perforation and the anterior septal mucosa on the left side for hemostasis and hydro dissection. A modified Killian incision of adequate length was performed 0.5–1 cm anterior to the septal perforation from the septal dorsum to the nasal floor. In order to facilitate elevation, the incision was made to the mucosal region where the underlying cartilage was thought to lie. Commencing the dissection from the edge of the perforation is contraindicated. Beginning from the incision, mucoperichondrial and mucoperiosteal flaps were advanced bilaterally to the upper and lower sides of the perforation endoscopically with the help of an elevator and were combined at the back of the perforation. The edge of the perforation was generally thin and adhered tightly due to previous surgery. The perforation edge and nearby mucosal parts were sharply dissected (Fig. 2).

**Fig. 3:**
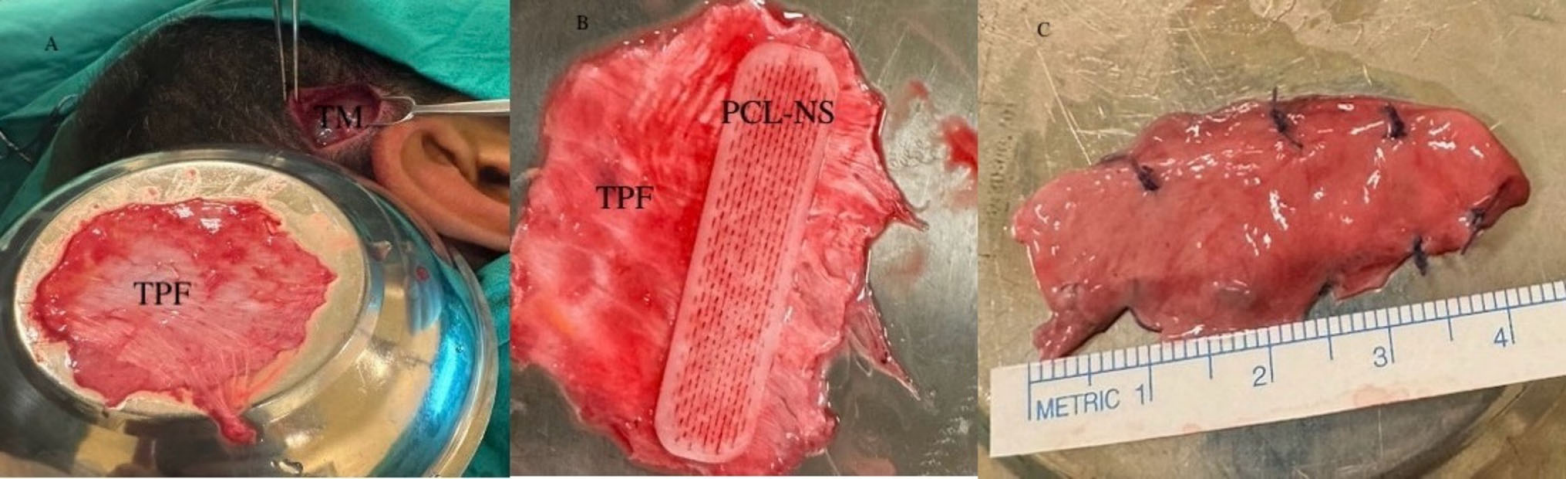
**A** Harvest of TPF off of deep temporal fascia. TPF, **B** A new construct is created according to dimensions of the perforation, **C** İnterposition graft consisting of a combined TPF and PCL NS. *TM* Temporal muscle, *TPF* Temporoparietal fascia, *PCL NS* Polycaprolactone nasal sheet

The aim of this sharp dissection was to prevent tearing and expansion of the perforation. A mucosal pocket was created around the flap for the graft placement. A graft material consisting of TPF and a PCL nasal sheet was used to close the perforation. The TPF was removed through a horizontal incision covering the middle part of the temporalis muscle (Fig. 3).

**Fig. 4:**
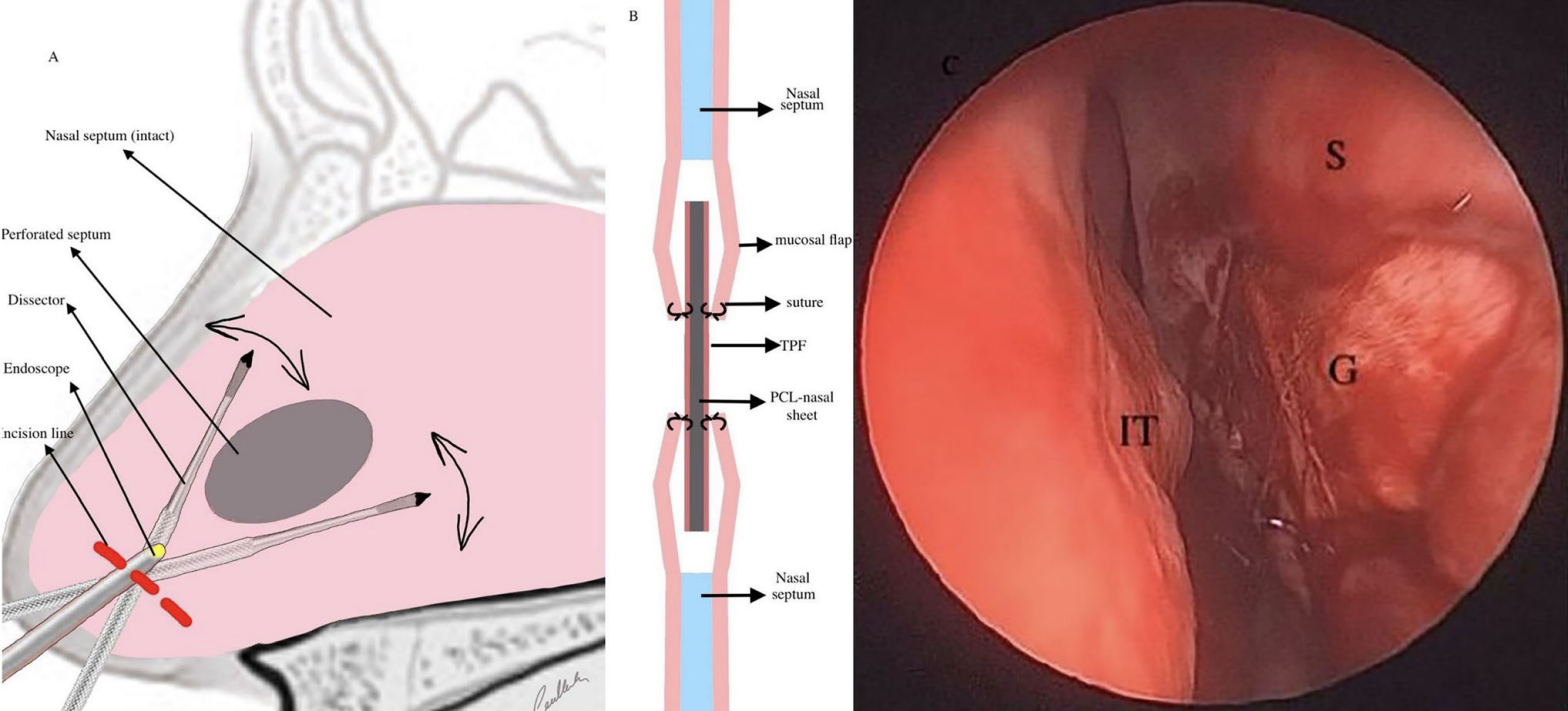
**A** Sagittal view of nasal septum (endoscopic incision and dissection). **B** Coronal view of graft placement. **C** Intraoperative view of perforation after graft placement. *S* Septum, *G* Graft, *IT* İnferior turbinate

Subcutaneous dissection was performed following the skin incision. The TPF was elevated from the superficial layer beneath the deep temporal fascia. Care was taken during this procedure to leave all the loose areola tissue. The PCL nasal sheet, the size of which was adjusted to exceed that of the perforation, was covered with sandwich-form TPF on both sides and was sutured with absorbable materials. The prepared sandwich graft was installed between the septum mucosal flaps such as to extend beyond the edges of the peroration (Fig. 4).

**Fig. 5:**
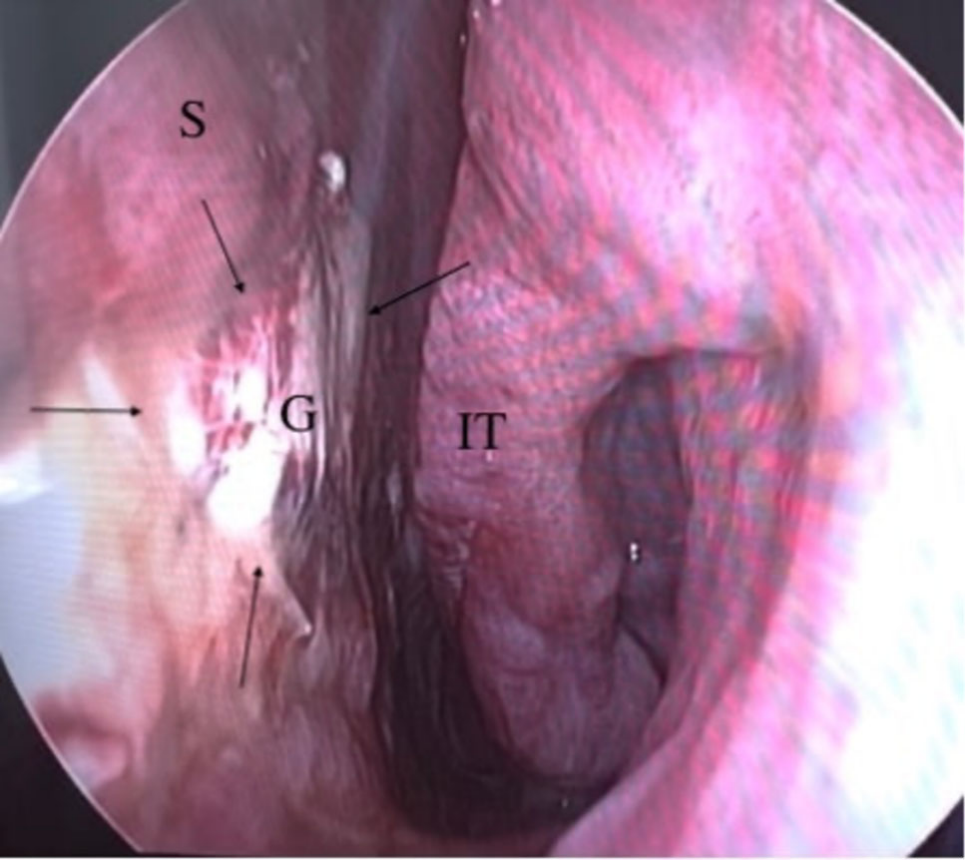
Postoperatively in the third week. *S* Septum *G* Graft, *IT* Inferior turbinate

The graft and septal mucosa were fixed by trans-septal or primary suturing with absorbable sutures. A silicon internal nasal splint was inserted into the bilateral nasal cavities. The silicon splints were removed postoperatively in the third week (Fig. 5).

## Results

Endoscopic septal perforation closure was performed on 15 patients, nine men and six women. The follow-up period ranged between 3 and 28 months, with a mean of 12 months.

**Fig. 6:**
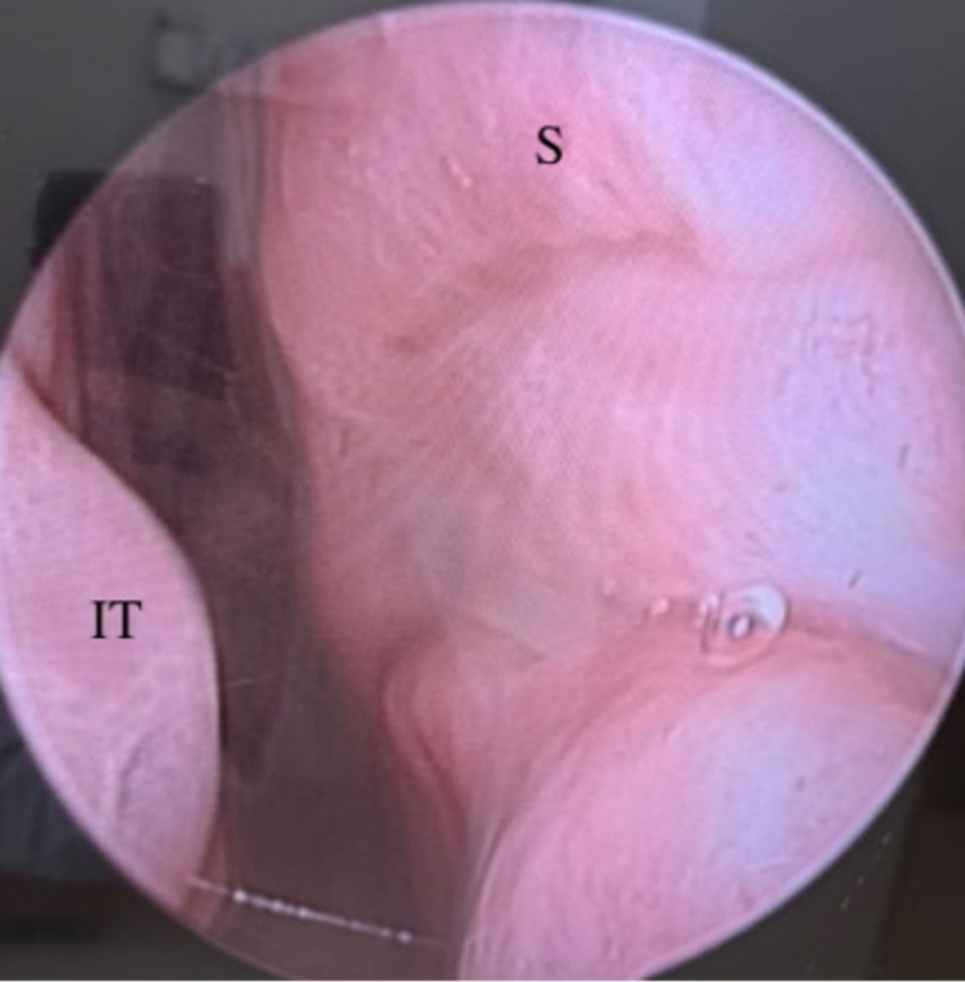
Perforation healed completely 3 months postoperatively. *S* Septum, *IT* İnferior turbinate

Complete closure was achieved in 13 of 15 patients (86.6%). Complete closure was observed in all the patients with medium-sized perforations and in four of the six with large-sized perforations (Fig. 6).

**Fig. 7:**
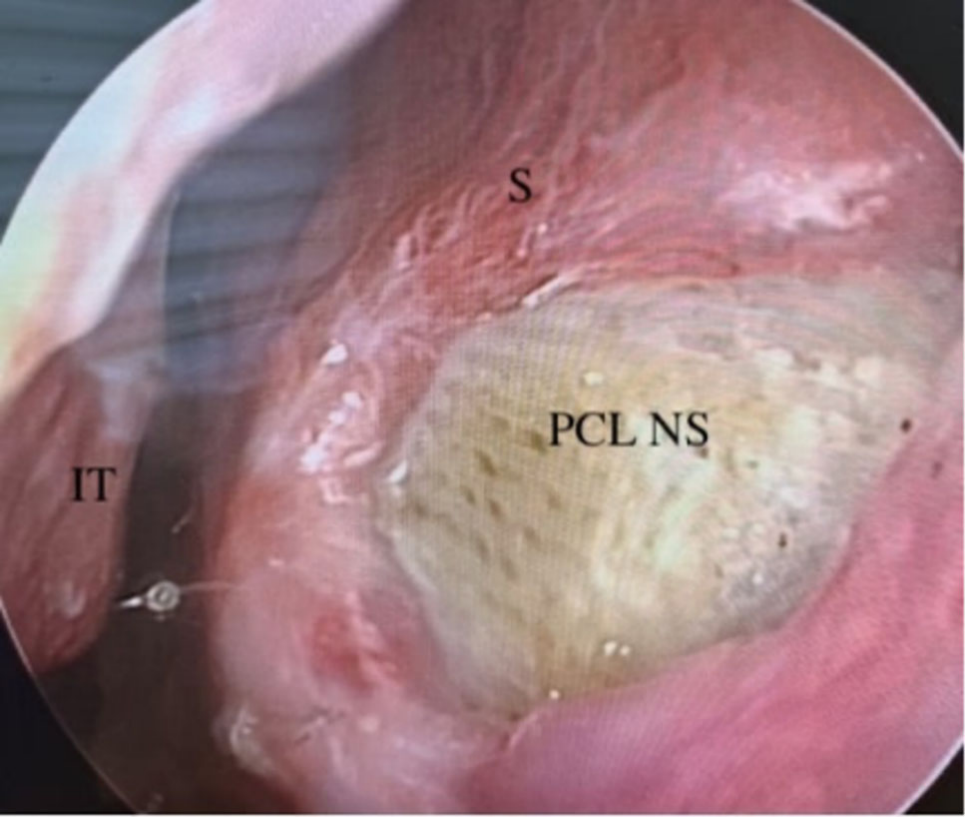
The diameter of the perforation was reduced, but there was no complete remucosalization. The stability of the PCL NS was preserved at a one year (Table 1 no:3). *S* Septum, *IT* İnferior turbinate, *PCL NS* Polycaprolactone nasal sheet

Absence of remucosalization and necrosis in the TPF graft was observed in two patients (Table 1, nos. 3 and 9). However, the stability of the PCL nasal sheet was preserved at a one-year follow-up (follow-ups are still continuing) (Fig. 7).

**Table 1:** Data of septal perforation repairment for 15 patients

No.	Age (yr)	Gender	Size, mm × mm	Etiology	Symptoms	Result
1	22	Female	10x11	Septoplasty	whistling sound, nasal crusting,	Success
2	27	Male	16x12	Septoplasty	nasal obstruction, nasal discharge	Success
3	59	Male	27x25	Septoplasty	nasal obstruction,	Failure
4	41	Female	18x12	Septoplasty	nasal obstruction, nasal crusting	Success
5	55	Female	10x13	Septoplasty	nasal crusting, whistling sound	Success
6	32	Female	15x13	Septoplasty	nasal obstruction, nasal discharge	Success
7	55	Male	16x14	Septoplasty	nasal obstruction, nasal crusting	Success
8	45	Male	21x15	Septoplasty	nasal obstruction	Success
9	26	Male	26x12	Septoplasty	nasal obstruction	Failure
10	29	Male	27x12	Septoplasty	nasal obstruction	Success
11	36	Female	25x15	Septoplasty	nasal obstruction, olfactory disorder	Success
12	39	Male	22x14	Septoplasty	nasal obstruction	Success
13	32	Male	15x10	Septoplasty	nasal crusting, whistling sound	Success
14	54	Male	15x12	Septoplasty	nasal obstruction, epistaxis	Success
15	30	Female	14x12	Septoplasty	nasal crusting, epistaxis	Success

No foreign body reaction or infection was observed in any patient. No complications also occurred in either the early or late postoperative periods. No crust formation was observed in the nasal cavity in any of the recovered patients. All data of septal perforation repairment for 15 patients are shown in Table 1.

## Discussion

Operations performed for nasal septal perforations entail a high degree of difficulty. To the best of our knowledge, this study represents the first reported retrospective case series of nasal septal perforation closure using a combination of bilateral TPF and a PCL nasal sheet. In this study, involving 15 patients and an average follow-up time of one year, the endoscopic sandwich graft technique emerged as effective in the repair of medium-sized and large-diameter iatrogenic NSP.

Whether or not patients with NSP experience uncomfortable symptoms depends on several factors, including the size and site of the perforation. In their study of 44 patients with symptomatic NSP, Khong and Leong observed a significant association between perforation-specific symptoms and small, anteriorly located perforations [15]. The most common symptom in NSP is nasal obstruction, seen in 65% of patients. This is followed by symptoms such as crusting (43%), nasal discharge (13%), a whistling sound (8%), and epistaxis (5%) [7]. In agreement with the literature, nasal obstruction was the most common symptom in the present study.

While no treatment is generally recommended for asymptomatic perforations, surgery is advised for patients with severe symptoms and/or insufficient relief after medical treatment. Numerous reconstruction techniques have been developed to repair NSP. Several surgical techniques are employed, depending on the dimension and location of the NSP and on the skill and experience of the surgeon. Since there is currently no standard protocol, deciding on the ideal NSP repair technique is problematic. The most frequently employed surgical technique for NSP repair is intranasal mucosal advancement flaps [2–5]. Highly successful results have been achieved with this technique. Ribeiro and Silva reported that full closure could not be achieved in only three out of 258 patients undergoing repair using the bilateral advancement flap technique with a closed septorhinoplasty approach for perforations between 1 and 3.5 cm in diameter [16]. Pedrosa et al. reported a 97% closure rate using the flap technique (perforation size > 1 cm in 88% of cases and < 1 cm in 12%) [5] . Villacampa et al. reported an 89% septal closure rate with the endonasal bilateral advancement flap technique in 37 patients [17].

The flap technique requires wide bilateral dissection sites for medium-sized and large perforations. This dissection is particularly difficult in patients with histories of nasal septal surgery. In addition, closing the perforation without tension is one of the principal conditions for success [6]. Thin, weakened septal mucoperichondrial layers are generally present in iatrogenic perforations, and this makes it difficult to elevate and advance the mucosal flap. However, wide dissection areas are not required with our endoscopic technique. The mechanism that permits closure of the perforation with an interposition graft alone without the use of a flap is the regenerative capacity of the mucosa, and the graft constitutes the basis of this closure process. A smooth surface on which the mucosa can proliferate is, therefore, placed between the mucosal flaps [6, 7] . The TPF, fascia lata, and rectus abdominis fascia, and materials such as autografts (septal cartilage, perpendicular bone, conchal cartilage, and costal cartilage) or a polydioxanone (PDS) plate capable of being entirely absorbed by the body via metabolization are employed as graft materials in this technique [3, 6, 7, 18, 19].

Attaching fascia grafts to the perforated area of the septum alone, correctly, and with adequate tension is difficult. PDS plate is frequently used as interposition graft material in NSP repair [20–23]. A PCL nasal sheet was employed to support the TPF graft in the present study. Park et al. reported that the use of three-dimensional printed PCL in nasal reconstruction facilitated surgery and was able to overcome the limitations of traditional alloplastic materials [24] . Some studies have reported that the use of three-dimensional printed PCL facilitated surgery in cases of nasal septal surgery and rhinoplasty, shortened the operative time, and prevented morbidity. In addition, researchers have suggested that a PCL sheet exhibits mechanical stabilization in nasal reconstruction and represents a safe and effective procedure with no need for excessive manipulation [12, 25, 26]. However, we have not encountered any previous studies concerning the use of PCL in NSP repair. TPF grafts are used as graft material in otological surgery, skull base repair, and ear reconstruction [27–29]. Some histological studies have shown that TPF possesses a well-organized, layered extracellular matrix consisting of both collagen and elastic fibers. This has led to its successful use as a material that increases cell migration and thus cellular regeneration [30].

NSP is most frequently iatrogenic in origin and has been found to derive from previous septoplasty or septorhinoplasty [31]. In their 56 case study involving the sandwich graft technique, Özer et al. reported closure in 93.3% of medium-sized and large perforations of idiopathic origin and in 81% of those with iatrogenic causes [6]. Kaya et al. reported an 86% success rate with a three-layer graft consisting of temporal fascia and conchal tissue in patients with small perforations less than 2 cm in size developing secondary to nasal septal surgery [18]. Chen et al. reported a 92.3% success rate in perforations 1–2 cm in size using an interposition graft, irrespective of etiological causes [7]. Similar success rates to those in the literature were achieved in the present study.

PCL has recently emerged as an alternative to autologous implants. PCL is a biocompatible material capable of being broken down biologically. It has also been shown to possess adequate thinness and mechanical support and to be capable of surgical manipulation [12–14]. PCL has also been reported to cause a low rate of foreign body reaction [32]. Some studies have also shown that it exhibits long-term stability (with bioabsorption over longer than two years) [33, 34]. No foreign body reaction or extrusion occurred in any patient in the current study. Nasal sheet stability was preserved even after an average of one year in two cases without remucosalization. In contrast to the flap technique, intense crusting may develop in non-mucosal closure techniques. In our clinical practice, silicon splints were kept in the nasal cavity for three weeks, and no crusting was observed following their removal. The microporosity of PCL promotes fibrovascular tissue formation without forming granulation tissue. However, the long-term outcomes after complete resorption of the PCL plate are not fully known and a long-follow-up clinical study is required for this [24]. Suboptimal positioning of the polycaprolactone (PCL) nasal sheet may lead to excessive pressure on the mucosal flap, potentially resulting in mucosal atrophy, extrusion of the nasal mucosa, and mucosal ulceration [12].

The PCL nasal sheet is a commercially available material. Its most important advantage is that it facilitates the installation and fixation of the TPF graft to the perforation area, as well as manipulation over the graft. It may also prevent the TPF graft from shrinking and protruding directly into the airway and, thus, any increase in surface area. This can produce a positive effect on remucosalization. Due to its porous structure, it can be fixed using trans-septal U sutures. Since the PCL nasal sheet employed in the present study was 1 mm thick, the sandwich graft did not cause any narrowing in the nasal airway.

We think that rather than an open rhinoplasty approach or the preparation of an intranasal flap in cases of iatrogenic NSP, the use of a three-layer interposition graft with the assistance of an endoscope for perforations of all sizes following mucosal elevation at the perforation edges facilitates surgery and shortens the operative time. Additionally, the long-term stability and smooth surface for the graft of the biologically absorbable PCL nasal sheet can be beneficial for remucosalization. Despite the promising results of the present study, its has limitations such as being a retrospective study with no comparison arm and the limited number of patients. One of the disadvantages of the technique is that the PCL nasal layer imposes an extra cost burden. Despite these limitations, this study may contribute to the literature as a simple, reliable method of TPF-PCL grafting in the NSP repair. Larger, controlled, and multicenter comparative studies with a large number of cases are needed to validate our findings.

## Conclusion

The endoscopic use of a three-layer interposition graft consisting of a PCL sheet and TPF for repair of medium-sized and large-sized iatrogenic NSP results in a high closure rate. The technique shows that the size of the perforation is not as important as the selection of the flap technique. The endoscopic sandwich graft can be safely applied and easily manipulated for iatrogenic NSP repair.

## Abbreviations

NSP: Nasal septal perforation
PCL: Polycaprolactone
TPF: Temporoparietal fascia
